# THE DUAL ROLE OF STUDENTS PURSUING A HIGHER DEGREE AND PROVIDING CARE TO THEIR CHILDREN AND FAMILY MEMBERS DURING THE COVID-19 PANDEMIC

**DOI:** 10.46827/ejes.v8i12.4018

**Published:** 2021

**Authors:** Forgive Avorgbedor, Helene Vilme

**Affiliations:** 1 School of Nursing, The University of North Carolina at Greensboro, Greensboro, NC, USA; 2 Department of Population Health Sciences, Duke University, School of Medicine, Durham, NC, USA

**Keywords:** caregivers, college students, COVID-19, pandemic

## Abstract

The objective of this qualitative study was to explore the psychological well-being of college students during the COVID-19 pandemic and highlight their experience as caregivers. A total of 1444 college students responded to the questionnaire on the impact of COVID-19 on their lives. Qualitative responses from 25 female and 7 male college students who serve as caregivers were analyzed. Data were assessed using thematic analyses. The analysis revealed that college students’ caregiving experiences impacted every aspect of their lives, including psychological health, academic performance, and career. Students who served as caregivers indicated that the mandated “Stay-at-Home” order resulted in homeschooling children, working from home, and attending school via online at home, limiting space for work, school, childcare, and homeschool. This led to increased stress, difficulty finding childcare, a drop in GPA for some, fear of delayed graduation, and financial distress.

**Conclusion:**

University students serving as caregivers were negatively affected during the pandemic. These individuals play a significant role in the workforce; therefore, rethinking resources and policies promoting their success as they serve their loved ones is in society’s best interest.

## 1. Introduction

The coronavirus disease (COVID-19) pandemic disrupted normal patterns of human lives (Matvienko-Sikar et al., 2020; Wardell et al., 2020), resulting in social isolation, school closures, and increased caregiving responsibilities (Pavalko & Woodbury, 2000; Pinquart & Sörensen, 2003; Prime, Wade, & Browne, 2020; Salami, Okoduwa, Chris, Ayilara, & Okoduwa, 2021). Caregiving involves the process of helping another person to meet physical, mental, emotional, and social needs (Hermanns & Mastel-Smith, 2012). Evidence indicates that younger adults are taking more caregiving responsibilities (Levine et al., 2005), suggesting that these caregivers could be in college pursuing higher education. Furthermore, these individuals who are potentially in college lack anticipatory guidance on the caregiving responsibilities (Baus, Dysart-Gale, & Haven, 2005), suggesting that the caregiving roles could significantly affect their physical and mental health (Trujillo, Perrin, Elnasseh, Pierce, & Mickens, 2016). A recent study found a significantly higher school-family conflict for working student parents compared to working students (Brauer & Foust, 2020). Before the pandemic, Denning et al. suggest the need to study college students balancing work, family, and school (Denning, Brannan, Murphy, Losco, & Payne, 2018) as this population is unique.

Although caregiver stress is well documented, very few studies have explored the stress of serving a dual role as a caregiver while pursuing a college degree. Therefore, the purpose of the study is to explore the impact of COVID-19 on individuals acting as caregivers while also pursuing a higher degree. Exploring the psychological wellbeing of students during the pandemic will highlight the experience of caregivers, which institutional organizations and governments can use to inform policies that strengthen the education pathway for this subset of the population that has dedicated themselves to serving others.

## 2. Material and Methods

### 2.1 Study Design

A mixed method approach was used to assess the impact of COVID-19 on college students. This manuscript explores the open-ended responses related to the impact of COVID-19 on individuals serving as caregivers while enrolled in a tertiary education program.

### 2.2 Recruitment and Data Collection

Between May and October 2020, students were recruited via university intranet systems, classroom announcements, and word of mouth using a standardized text indicating the purpose of the study, eligibility criteria, data collection procedures, and the survey link.

### 2.3 Ethical Approval and Consent

This study was approved by Duke University and the nine collaborator’s colleges and universities. Informed consent was obtained via Qualtrics before students were able to access the questionnaire.

### 2.4 Data Analysis

The coding and theme identification processes for this manuscript were guided by Braun and Clarke (2006) six steps process: 1): familiarizing with data; 2) generating initial coding; 3) Searching for themes; 4) Reviewing of themes; 5) Defining and naming of themes; 6) Reporting the findings. The co-authors, FA and HV served as the coders for this manuscript and analyzed the student’s open-ended responses using NVivo. Concerning familiarization with the data, FA and HV reviewed the open-ended responses by reading the responses multiple times, jotting down initial impressions independently to reduce analysis coding bias. To generate the initial codes, FA and HV independently searched for recurring patterns to generate the initial coding across the entire data set. Once the initial coding was generated, it was subsequently refined with input from all co-authors. Afterward, FA and HV independently searched for themes by reviewing the responses of the open-ended questions searching for meaningful patterns. Following the identification of themes, FA and HV independently read the text associated with each theme, checking the themes relevance in relation to the codes extracted and across the entire data set. Ongoing analysis to refine the themes involved FA and HV meeting to identify the ‘essence’ of each theme by discussing how each theme fit with each other and the purpose of the study. The final step consisted of reporting the present manuscript findings by selecting compelling extracted examples of the coded text for each theme, presented below.

## 3. Results

### 3.1 Sample Characteristics

Out of the 2,035 students in the US who participated in the study, 1,444 responded to an open-ended question about students’ experiences with COVID-19. A total of 32 students, including both men and women, disclosed the overall impact of COVID-19 related measures such as school closures on their academic work and increased responsibility of providing care to family and friends. The participants mean age was approximately 35 years. Table 1 shows the detailed description of the participants included in this manuscript.

As shown in Table 2, we identified seven themes regarding the dual role of schooling and providing care to friends and family during the pandemic, including providing care and childcare; needs and difficulty.

### 3.2 Psychological Impacts on Students Resulting from Caregiving Roles

The participants in this study expressed their experience pursuing higher education and being responsible for family and friends’ care during the pandemic. They indicated that being a caretaker while pursuing a college degree was difficult, and some expressed that it had a psychological toll, impacting eating habits and academic performance.

“As a parent of 3 school-aged children, a full-time employee in a university research setting, and a graduate student in public health, COVID-19 has touched every part of my life. At the start of the pandemic, the whirlwind of having 3 kids in online school, then applying for rapid-response grants at work related to COVID-19, all while constantly analyzing the pandemic in my Epidemiology classes, I felt I could not escape COVID. It took a toll on me psychologically and in turn, my eating habits worsened and I drank more than a healthy amount of alcohol.”

(40, Female, Graduate student, Midwest)

“It has been stressful and with two children now at home full time, it is more difficult to complete school work and will likely result in my taking a decreased course load.”

(41, Female, Graduate student, South)

“As a single mother, who works full time, has two first graders and is taking grad school courses, it has been incredibly stressful. I managed to finish the spring semester once it transitioned to online. I struggled with whether to continue classes this fall given the challenges with having two young school age children who are also home full time virtually while I’m also trying to get my own work done. I decided to try, but it has been difficult. I worry I may need to pause classes which would delay my graduation and I fear if I lose momentum now, I may not end up re-engaging with my studies. If my university decides to go back to on-campus instruction I may also need to consider dropping classes as I am the sole parent to my kids and I have asthma and I am a caregiver for my 88 year old grandmother. I need to remain healthy.

(37, Female, Graduate student, Midwest)

“I work in the medical field full time as well as taking college courses and trying to homeschool my 4 kids as a single parent. This has been beyond brutal and depressing. I am overworked, underpaid, and struggle trying to educate my kids where the system has failed them in many ways. My own grades are suffering, and I barely make it by financially.”

(40, Female, Other, Midwest)

“My GPA had slid from a 3.9 to a 3.6 and I handed in my first late assignment ever, this semester. I am having a hard time juggling everything”

(48, Female, Graduate student, Midwest)

### 3.3 Social Distancing is a Source of Stressors for Student Caregivers

Social distancing and isolation are the hallmarks of public health measures to mitigate the rapid spread of COVID-19. The participants indicated that the public health measures, especially isolation, were the source of stressors.

“The biggest stressor has come from working from home. Everything moved to this one space- work, childcare, school.”

(34, female, Graduate Student, South)

“It has been stressful and with two children now at home full time.”

(41, Female, Graduate student, South)

### 3.4 Financial Distress Concerning Meeting Basics Needs while Serving as a Caregiver and Student

As public health measures were put in place, parents were responsible for providing care to their friends and family and bore the responsibility of keeping their families safe. Some participants described the overall effects of the pandemic on their entire lives, including the impact it had on their finances and ability to meet basic needs.

“We have been faced with many challenges. Then between my pay being cut, my husband getting laid off, we have had to turn to credit cards in order to purchase food.”

(40, Female, Senior, Midwest)

“My insomnia has gotten worse. I lost 15 lbs because I have lost my appetite. I don’t do well in this environment, and my grades dropped. Very scary. Worried about being safe, food, jobs, and household items. COVID-19 has put a strain on my personal life and school performance. Like stated in a previous question, my children’s daycare was closed so I am now home with them full time with no help.”

(20, Female, Sophomore, South)

### 3.5 Increased Responsibilities as a Caregiver and a Student

As the family responsibility increased, the participants’ roles shifted; they embraced the caregiving role and deemed their academic work extra. The sudden change in the home setting was disruptive and added additional stress to the home. The responsibility shifts have also impacted the ability of student caregivers to complete their work and focus on their studies.

“It has been a rough transition for me because I am the family’s day-to-day caretaker, but I know others who have had it much worse. It is hard to concentrate on schoolwork and motivate myself because of my family and constant chores. I feel like school has become so much more stressful and burdensome.”

(20, male, Sophomore, South)

“It has been stressful. My mother and teenage daughter have become clinically depressed and anxious. My two college Junior/seniors came back to my house. I am immune-compromised and my spouse does not take COVID seriously enough. I have had trouble procuring the basic necessities at times, which is exacerbated by not being able to shop in public.”

(48, Female, Graduate student, Midwest)

Participants expressed needing childcare and difficulty finding childcare. The participants required childcare to focus on their academic work.

### 3.6 Needs for Childcare due to School Closures

“The strain of parenting during a pandemic while going to school, and the differential stress on mothers versus fathers. Time! My children are at home full time and managing childcare with my responsibilities is basically impossible.”

(41, Female, Graduate student, Midwest)

“My children’s daycare was closed so I am now home with them full time with no help. They are 1 year old twins, so I have little time to study or complete my courses which reflected in my grades for the semester.”

(20, Female, Senior, South)

“Work and classes are difficult without childcare”

(34, Female, Graduate student, South)

“I’m a stay at home parent to a toddler and infant and I typically need to get away to work, which I haven’t been able to do since Covid started”

(34, Female, Graduate student, Midwest)

“Work and classes are difficult without childcare. Homeschooling my children for the end of the spring semester was even more difficult, but we got through it.”

(34, Female, Graduate student, South)

### 3.7 Difficulty Finding Childcare

The participants disclosed their desire to obtain childcare but were met with difficulty finding childcare. Lack of childcare negatively impacted the caretakers.

“Childcare is not easy to find during a pandemic.”

(32, Female, Graduate student, South)

“Difficulty with childcare so I can still work and complete my classes. Stressful.”

(42, Female, senior, Midwest)

## 4. Discussion

The objective of this manuscript was to explore the impact of COVID-19 on individuals serving as caretakers/caregivers while also pursuing a higher degree. The themes that emerged were psychological toll, isolation as a source of stressors, financial distress and concern meeting basics needs, increased responsibilities, need for childcare, and difficulty in finding childcare.

Although the participants combined their caregiving role with their academic work, the pandemic exerted a psychological toll leading to worsened eating habits and academic performance. One of the unhealthy habits linked to COVID-19 is poor dietary health behaviors (Khubchandani, Kandiah, & Saiki, 2020; Werneck et al., 2020), which is linked to overall health and wellbeing (Mattioli, Puviani, Nasi, & Farinetti, 2020). For students who are also caregivers, physical and mental health is paramount to provide care and perform well in their studies.

As with a decline in mental health and mounting pressure from their caregiving role, the participants’ academic performance was lagging, resulting in decrease GPAs. GPA plays a role in individuals furthering their educations (Hen & Goroshit, 2014; Ibrahim & Wah, 2020). Research indicates that level of education is not simply associated with future earnings but also with an individual’s health status (Zajacova & Lawrence, 2018). Thus, there is a need to consider the impact of the pandemic on students serving as caregivers when creating infrastructure, policies, and procedures to handle future pandemics.

One of the first public health measures and a necessary action early in the pandemic was social or physical distancing (*Advice for the Public*, 2020). The measures resulted in social isolation and the inability to socialize, see friends, and do activities that help cope with stress. Similar to Devaraj and Patel (2021) as well as Wang et al. (2020) findings, participants in this study were affected psychologically by the preventive measures in the US that restricted mobility early in the pandemic. Furthermore, the participants reported that isolation was a stressor for them because everything (school, work, and childcare) was designated to a single space, the home. These activities taking place in an area that was not originally designed to accommodate all these activities affected the psychological health of the caregivers during the pandemic. Research has shown that caregivers have a higher level of stress than non-caregivers (Davidson et al., 2020; Pinquart & Sörensen, 2003) Poor psychological health has been linked to physical inactivity (Chen et al., 2020) and poor health maintenance and coping (Wardell et al., 2020). The mental wellbeing of caregivers is paramount to the well-functioning of the family unit (Prime et al., 2020) and the ability of the caregiver to meet the needs of the care recipients (Kohls, Baldofski, Moeller, Klemm, & Rummel-Kluge, 2021).

Furthermore, the effect of the pandemic on the caregivers resulted in financial distress and concern for meeting basic needs. The economic distress resulted from the loss of jobs by the participants or their spouse or a family member. During the earlier part of the pandemic, evidence suggested that women were more likely to lose their jobs than men (Alon, Doepke, Olmstead-Rumsey, & Tertilt, 2020; Kabeer, Razavi, & van der Meulen Rodgers, 2021). Moreover, some students, particularly women, had to choose caregiving over employment because schools were closed, and they had to take up their children’s education (Bahn, Cohen, & van der Meulen Rodgers, 2020; Woskie & Wenham, 2021). As demonstrated in the results of this study, women were at the forefront of caregiving, and they were disproportionately part of the unpaid domestic work pre-pandemic (Revenson et al., 2016; Russell, Hutchison, Tambling, Tomkunas, & Horton, 2020). The pandemic magnified the burden women have carried as part of day-to-day activity. Thus, the pandemic has created the opportunity for society to reflect on the role of women and provide policies and resources to reduce the burden on women, especially while they manage the dual role of students and caregivers.

Additionally, the participants also expressed their desire to find childcare during the pandemic. They reported that childcare was not easy to find and that it was an extra responsibility that impacted their academic performance. Students’ caregivers may lack coping skills to deal with increased responsibilities leading to poor academic performance (Brauer & Foust, 2020; Champlain, 2012). The pandemic has provided the rationale for society to provide community resources that can help alleviate some of the burdens students face while pursuing higher education and meeting all the demands that society imparts on them.

### 4.1 Limitations

The experience of the caregiving role presented in this paper is based on the question relating to the overall impact of COVID-19 on college students. The parent study questionnaire did not directly ask about the caregiving role as a college student, hence the responses related to this topic were limited. However, the issues relating to caregiving roles and academic work significantly impacted the participant’s lives, thus, future studies need to explore the issue further.

### 4.2 Recommendations

College students should be aware that the dual role as a student and caregiver and has potential impacts on their physical and mental health. Society must provide resources for students to effectively perform both roles and maintain their mental health.

## 5. Conclusions

The caregiver burden is well documented in studies. However, the dual role of caregiving and pursuing higher education has yet to be explored fully. Students are attending school while working, providing care for their children and sometimes family members. The added challenges of navigating all of their responsibilities during the pandemic negatively affected all aspects of students’ lives. College students are the backbone of the future workforce, and rethinking resources and policies that can promote their success is in the best interest of society.

## Figures and Tables

**Table 1:** Characterizing of college students study participants

Characteristic	*n* (%) or *M* ± *SD* [*Med*] (*Min, Max*)
Age (years)	35 ± 8.4 [34.5] (20, 51)

Gender	
Male	7 (22)
Female	25 (78)

Race/Ethnicity	
Asian	2 (6.3)
Black of African American	2 (6.3)
Middle Eastern	1( 3.1)
Multiracial	1 (3.1)
Other	1 (3.1)
White	25 (78.1)

Education	
Graduate students	22 (68.8)
Juniors	1 (3.1)
Sophomores	3 (9.4)
Seniors	5 (15.6)
Other	1 (3.1)

Education Classification	
Full-time	18 (56.2)
Part-time	14 (43.8)

**Table 2:** Themes, Codes, and Exemplar quotations

Themes	Code s	Exemplar quotations
Psychological toll, isolation as a source of stressors, financial distress, meeting basics needs, and increased responsibilities.	Caretaker/caregiver	*“As a mom, this has been a difficult situation. Between trying to explain things to my children, trying not to let them be scared, then working full time, plus helping them with their online schooling*.” (40, Female, Senior, Midwest) *“I am a caretaker for someone with dementia, so it has been very difficult being stuck in the house for months on end with someone in that constant mental state. It is hard to prioritize school when your mental health is so greatly impacted.”* (28, Female, Graduate student, Midwest)
Need for childcare, and difficulty in finding childcare	Needs childcare	*“I’m a single parent, which was not a question. Having a toddler in the house without childcare impacts my ability to complete my coursework and do my job responsibilities.”* (34, Female, Graduate, South)
		*“I am a single mom with two young kids, so the debate about them going back to school or not has been the biggest stressor. Childcare is not easy to find during a pandemic.” (32, Female, Graduate Student)* *“Having a toddler in the house without childcare impacts my ability to complete my coursework and do my job responsibilities.” (34, Female, Graduate student, South)*
	Difficulty finding childcare	*“Difficulty with childcare so I can still work and complete my classes.” (42, Female, Senior, Midwest)*
